# Multi-Modal Characterization of Monocytes in Idiopathic Pulmonary Fibrosis Reveals a Primed Type I Interferon Immune Phenotype

**DOI:** 10.3389/fimmu.2021.623430

**Published:** 2021-03-05

**Authors:** Emily Fraser, Laura Denney, Agne Antanaviciute, Karl Blirando, Chaitanya Vuppusetty, Yuejuan Zheng, Emmanouela Repapi, Valentina Iotchkova, Stephen Taylor, Neil Ashley, Victoria St Noble, Rachel Benamore, Rachel Hoyles, Colin Clelland, Joseph M. D. Rastrick, Clare S. Hardman, Nasullah K. Alham, Rachel E. Rigby, Alison Simmons, Jan Rehwinkel, Ling-Pei Ho

**Affiliations:** ^1^MRC Human Immunology Unit, Weatherall Institute of Molecular Medicine, University of Oxford, Oxford, United Kingdom; ^2^Department of Immunology and Microbiology, School of Basic Medical Sciences, Shanghai University of Traditional Chinese Medicine, Shanghai, China; ^3^Department of Computational Biology, Weatherall Institute of Molecular Medicine, University of Oxford, Oxford, United Kingdom; ^4^Single Cell Genomics Facility, Weatherall Institute of Molecular Medicine, University of Oxford, Oxford, United Kingdom; ^5^Department of Thoracic Imaging, Oxford University Hospitals NHS Foundation Trust, Oxford, United Kingdom; ^6^Immunology Therapeutic Area, UCB Pharma, Slough, United Kingdom; ^7^Nuffield Department of Surgical Sciences and Oxford NIHR Biomedical Research Centre, University of Oxford, John Radcliffe Hospital, Oxford, United Kingdom; ^8^Oxford Interstitial Lung Disease Service, Oxford University Hospitals NHS Foundation Trust, Oxford, United Kingdom

**Keywords:** monocytes, lung, fibrosis, idiopathic pulmonary fibrosis, macrophages

## Abstract

Idiopathic pulmonary fibrosis (IPF) is the most severe form of chronic lung fibrosis. Circulating monocytes have been implicated in immune pathology in IPF but their phenotype is unknown. In this work, we determined the immune phenotype of monocytes in IPF using multi-colour flow cytometry, RNA sequencing and corresponding serum factors, and mapped the main findings to amount of lung fibrosis and single cell transcriptomic landscape of myeloid cells in IPF lungs. We show that monocytes from IPF patients displayed increased expression of CD64 (FcγR1) which correlated with amount of lung fibrosis, and an amplified type I IFN response *ex vivo*. These were accompanied by markedly raised CSF-1 levels, IL-6, and CCL-2 in serum of IPF patients. Interrogation of single cell transcriptomic data from human IPF lungs revealed increased proportion of CD64^hi^ monocytes and “transitional macrophages” with higher expression of CCL-2 and type I IFN genes. Our study shows that monocytes in IPF patients are phenotypically distinct from age-matched controls, with a primed type I IFN pathway that may contribute to driving chronic inflammation and fibrosis. These findings strengthen the potential role of monocytes in the pathogenesis of IPF.

## Introduction

Idiopathic pulmonary fibrosis (IPF) is the most severe form of chronic fibrotic lung disease (1). The fibrosis is progressive, and median survival is only five years from diagnosis. Repeated but minor insults to the alveolar epithelium are thought to lead to a disproportionate repair response by fibroblasts and other mesenchymal cells (2).

Research has focused more intensely on the abnormal repair response, and less on the drivers of chronic fibrosis. A recent study suggest that monocytes may be linked to progression of fibrosis in IPF, since increased levels in IPF patients were correlated with poorer survival (3). However, no further immune phenotyping data on these monocytes were available to guide further mechanistic studies.

Monocytes are a heterogeneous group of immune cells with significant plasticity in their phenotype and function (4). In murine studies, monocyte-derived macrophages (as opposed to resident alveolar macrophages) appear to be critical for the development of lung fibrosis and potentially also for its resolution (5–7). In organs other than lungs, animal studies have described the ability of monocytes to enhance myofibroblast proliferation in cardiac muscles after infarction (8), and the generation of immature monocytes in the bone marrow correlates with pro-fibrotic features after an injurious stimuli (7). However, despite increasing evidence for the role of monocytes in fibrosis in bleomycin murine models (widely acknowledged as imperfect models of the human disease), there is still limited human data to support a role for monocytes in the pathogenesis of IPF.

In this study, we examined the immune phenotype of monocytes in the blood and lungs of IPF patients, and explored if they showed abnormalities that might contribute to the pathogenesis of IPF. In blood, we found that monocytes from IPF patients were phenotypically distinct compared to age-matched controls, displaying an increased expression of CD64 protein and an amplified type 1 IFN response when stimulated *ex vivo*. Interrogating single cell transcriptomic data in IPF lung explants, we found increased proportion of monocytes and “transitional” (less mature) macrophages that re-capitulated the features of circulating blood monocytes.

## Methods

In the first part of the two-year project, IPF patients and age-matched healthy controls were prospectively recruited from the Oxford Interstitial Lung Disease Clinical Service in 2017; aiming for a pragmatic number of 50 IPF and 50 healthy control in the year-long study. Within the study year, 37 IPF patients fitted the inclusion criteria (see below) and 28 age-matched healthy controls were recruited. Each recruiting session included a few IPF patients and at least one heathy control sampled together, to reduce disease vs control batch effect. This influenced the numbers of patients and controls. Blood was withdrawn to isolate peripheral blood mononuclear cells (PBMCs) and CD14 monocytes [by positive bead selection (see below)] and used fresh for immune phenotyping by multi-colour flow cytometry, qPCR or bulk RNA sequencing. In the second half of the year serum were also collected to explore the cause for emergent findings. In patients where a CT scan was performed clinically within a month of the blood sampling, the CT was scored for the amount of fibrosis according to methods described below (9). We analyzed the data at the end of 2017, and with the finding of increased CD64 expression in monocytes, we next tested the possibility that this was related to perturbation in type I interferon related genes in monocytes. This was done using bulk RNA sequencing of monocytes from three well characterized IPF patients and controls. A further cohort of IPF patients (fulfilling the same inclusion and exclusion criteria) were then recruited from the clinical service to test the expression and functionality of interferon stimulated genes (ISG). Finally, to complement these studies, we interrogated the single cell transcriptomic data from lung cells derived from 4 IPF patients and 6 healthy controls deposited by Reyfman and colleagues (10). These are described in detail under relevant parts of Results.

### Patients and Controls

All patients with IPF as diagnosed by multidisciplinary team discussion using criteria defined by the 2011 American Thoracic Society Statement (11) were approached for recruitment. Patients also had to have a “definite” or “probable” usual interstitial pneumonia (UIP) pattern on thoracic CT scans according to the pre-2018 ATS statement (11), be non-smokers for five years, and did not have concomitant lung or cardiac diseases, immune conditions, or any cancers. All patients with emphysema on thoracic CT scans that were more than 50% of the amount of fibrosis [as reported by our interstitial lung disease thoracic radiologists (VSN or RB)]; and those who reported change in symptoms or infective symptoms in the two weeks before sampling were excluded.

Age matched healthy controls were recruited from pre-assessment clinics and volunteers at the University throughout the study. At least one healthy volunteer sample were processed at the same time as IPF samples, to reduce impact of batch sampling. Volunteers were asked a series of question about their health, smoking history and medications they took. Those with formal health conditions other than essential hypertension, and those who are current smokers, on medications other than one diuretic anti-hypertensives were excluded.

The study had ethical approval from the local and UK national ethics committee (14/SC/1060 from the Health Research Authority and South Central National Research Ethics Service).

### CT Score for Fibrosis

A previously published CT scoring system was used to quantify lung fibrosis (9). Briefly, six anatomically-defined axial sections of the thoracic HRCT were selected for analysis. The proportion of honeycombing, reticulation, traction bronchiectasis, and ground glass opacification admixed with traction bronchiectasis (taken to signify that the ground glass opacification was fine fibrosis) within each section were scored to the nearest 5%. By protocol, ground glass changes without admixed traction bronchiectasis were not included in this score; though as expected in IPF, this was hardly observed in the CT scans of our patients. The total fibrosis score is then calculated by adding the scores of all the individual fibrotic scores.

### Isolation of PBMCs and Monocytes

Blood samples were collected in lithium heparin (Greiner Bio-one) and processed fresh without storage. Peripheral blood mononuclear cells (PBMCs) were extracted by Lymphoprep™ (Axis-Shield) density gradient separation. Monocytes were isolated from PBMCs by positive selection using anti CD14 microbeads (Miltenyi Biotec) according to the manufacturer’s instructions. CD14^+^ monocyte purity was assessed by flow cytometry (CD3, CD19, CD15, CD16, and CD14). Only samples with purity of greater than 98% were used.

### Serum Preparation and Soluble Mediator Analyses

Serum were isolated and stored at -20°C for batch testing for selected soluble mediators using human magnetic bead Luminex multi-analyte assay following the manufacturer’s protocol (Bio-techne custom analyte mix). Results were obtained with a Bio-Plex 200 System (Bio-Rad). CSF-1 was measured by standardized sandwich ELISA (R&D Systems).

### Flow Cytometry

All antibodies were purchased from Biolegend. Cells were incubated with antibodies against surface antigens for 20 min at 4°C and fixed with Stabilising Fixative (BD) prior to acquisition. Assay standardization was performed using Rainbow Calibration beads (Thermofisher). Cells were acquired using a LSRFortessa™(BD) and data was analyzed using Flowjo v10 software (Tree star, Inc) and FACSDiva™(BD).

### Bulk RNA Sequencing

Circulating monocytes were isolated by CD14^+^ Macsbead positive selection and RNA extracted using RNeasy Mini Kit (Qiagen) as described above. RNA integrity number (RIN) exceeded 9 for all samples measured by 2100 Bioanalyser (Agilent). RNASeq libraries where prepared using Smartseq2 as described by Picelli et al. (12). Resulting libraries were converted to Illumina compatible libraries using Illumina Nextera XP kit, as per manufacturers instructions, and sequenced using NextSeq 500(Illumina) (single end, 75 bp unpaired sequencing). Sequencing depth was 30 million per sample.

FASTQ files were generated and quality of raw sequencing reads was initially assessed using fastQC (13). Poor quality bases (<20) and technical sequences were trimmed using Cutadapt software and reads were subsequently aligned using STAR aligner (14) against the human genome hg38 assembly. Non-uniquely mapped reads were discarded and gene expression levels were quantified as read counts using the featureCounts function (15) from the Subread package (16) with default parameters. There were 27.4–38.3M final uniquely mappable reads per sample. The read counts were normalized using library size factors to account for differences in sequencing depth and/or RNA composition, calculated using the median ratio method, as described in Anders and Hubert (17). Differential expression performed using the DESeq2 R package (18). R package clusterProfiler (19) was used to perform enrichment studies on differentially expressed genes and GSEA on all genes between the two groups, using gene sets from GO and Reactome pathways.

### Real-Time PCR

For quantitative gene expression in the type 1 IFN signaling studies, real-time PCRs for genes were performed using TaqMan Fast Advanced Master Mix (Applied Biosystems) with TaqMan primer/probe sets for human genes [*STAT1, IRF7, MX1, MX2, ISG15, IFI44L, IFI27, USP18, OASL, RASD2, IFNB1, IFNA1*, and *FCGR1A* (CD64)]. Real-time PCRs were performed on a QuantStudio 7 Flex Real-Time PCR System and threshold cycle (CT) values were determined from duplicate reactions using QuantStudio software (Thermo Fisher Scientific).

### IFN-β Stimulation of Cultured Monocytes

CD14 monocytes plated at 200,000 cells per well in 96 well flat bottom plates in 200 μl complete RPMI-1640 media supplemented with 2 mM L-glutamine, 100 IU/ml penicillin/streptomycin and 10% heat-inactivated fetal calf serum (Sigma-Aldrich). Monocytes were stimulated for 18 h with 100 U/ml recombinant human IFN-β1 (R&D systems) and processed for RNA extraction.

### Statistical and Single Cell Transcriptomic Analysis

For comparison between two groups, distribution of data was tested first with D’Agostino & Pearson normality test. Comparison of normally distributed data was performed using Student’s t test, and with Mann-Whitney Rank Sum test where data were not normally distributed. Correlation for normally and non-normally distributed data was performed using Pearson and Spearman’s Rank Order correlation tests, respectively.

Single cell gene expression matrices for each sample were downloaded from GEO (accession: GSE121611) in hdf5 file format and read into R for further processing. For each sample, DropletUtils R (20) package was used to perform cell calling. Barcodes determined as non-empty (< 1% FDR) were retained for further processing. Additionally, poor quality cells (> 10% mitochondrial RNA and < 500 detected genes) were filtered out. R packages Seurat (21) and liger (22) were used for subsequent analysis. Data from individual samples were integrated and batch correction performed using “liger” algorithm. First, data were normalized and highly variable genes were selected using “selectGenes” function in liger, with parameters var.thresh=c(0.1, 0.875) and num.genes=2000. The normalized expression values for highly variable genes were scaled prior to integration. The optimum number of factors, k, was first determined by computing the median Kullback–Leibler divergence from uniform for cell factor loadings (“suggest” function, default parameters) followed by optimization of the penalty parameter lambda (function suggestLambda, default parameters). Shared factors between datasets were then computed and quantile aligned (functions optimiseALS and quantileAlignSNF). Louvain cluster analysis was carried out in the aligned factor space, with the resolution parameter set to 0.4. Clusters were annotated to broad cell types based on the cluster marker profiles reported by Reyfman et al. (10). Aligned single cell clusters were visualized as tSNE embeddings.

To confirm the cell annotation, we also downloaded data and cell type annotations provided by Adams et al. (23) (GEO accession: GSE136831), processed the data as described above and then used this as a reference to transfer cell type annotations to Reyfman et al. dataset. Using R package Seurat “FindTransferAnchors” and “TransferData” functions, we computed prediction probability matrix for each cell for each Adams et al. cluster and assigned the likeliest cell type label as that of the maximum probability class.

R package “AUCell” (24) was used to score individual cells for interferon signaling pathway activities. First, we subset the expression matrix to retain only myeloid cell cluster cells and removed genes expressed in fewer than 10 cells. For each cell we used the expression matrix to compute gene expression rankings using AUCell_buildRankings function (default parameters). Interferon signaling related gene sets (Gene Ontology and Reactome pathways) were downloaded from the Broad Institute website, as part of the MSigDB gene sets (25). Gene sets were then used to score each cell where, for each gene set and cell, area-under-the-curve (AUC) values were computed (AUCell_calcAUC function) based on gene expression rankings, where AUC values represent the fraction of genes within the top-ranking genes for each cell that are defined as part of the pathway gene set.

## Results

### IPF Patients Showed High Serum CSF-1, CCL-2, and IL-6 Levels, and Increased Blood CD64^hi^ Monocytes that Correlated With Extent of Lung Fibrosis

Patient demographics for this part of the study are found in **Supplementary Table 1**. Data were analyzed for all IPF patients (n=37) vs age-matched healthy controls (HC) (n=28), and also after dividing patients into those on Pirfenidone treatment compared to those without.

**Figure 1:**
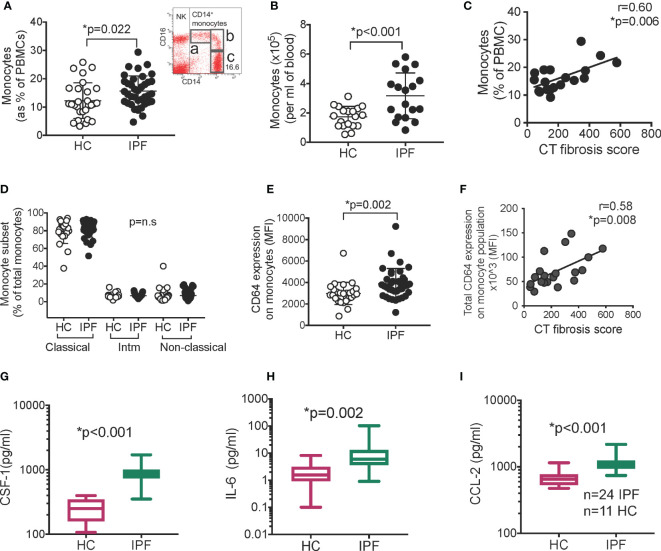
Monocytes and serum CSF-1, IL-6, and CCL-2 are raised in IPF. **(A)** Monocytes levels in IPF (n=37) vs healthy controls/HC (n=28) as % of PBMC. Inset- gating for monocytes. FACS plot was gated on all live PBMCs. “a”- non-classical monocytes, “b” –intermediate monocytes, “c” – classical monocytes; “NK” – natural killer cells **(B)** Monocytes as absolute number in blood (n=18). **(C)** Monocyte levels against amount of lung fibrosis on contemporaneous thoracic CT scans **(D)** Monocyte subsets in IPF and HC “Intm” – intermediate. Populations shown in **(A)**. **(E, F)** Expression of CD64 (n=37) determined by flow cytometry; and correlation between CD64 expression on monocytes and CT fibrosis score (n=19). p values derived using Student t test for normally distributed data, or Mann Whitney Rank Sum test if not normally distributed. Correlation analyzed using Pearson correlation test. **(G–I)** Levels of CSF-1, IL-6, and CCL-2 in serum measured with Luminex technology (n=24 IPF; n=11 healthy controls, HC). Y axis in for **(G–I)** is expressed as log 10. Box plot is median+/1 interquartile confidence interval, whiskers show minimum and maximum values. p values calculated using Mann Whitney Rank Sum test for **(G–I)**.

Monocytes were defined as CD14^lo-hi^ CD16^neg-hi^ cells (**Figure 1A**). Levels were higher in IPF patients compared to HC (as % of PBMCs and absolute numbers (per ml of blood; performed in a subset of patients) (**Figures 1A, B**) [16% (5) v 12% (6); IPF vs HC; p=0.022 and 3.2x10^5 (1.6) v 1.7x10^5 (0.7)/ml; p<0.001 respectively; mean(S.D.)]. There was no difference comparing patients on Pirfenidone to those who were not (**Supplementary Figures 1A, B**).

Monocyte levels correlated positively with the extent of fibrosis in the patients’ lungs, as measured on high resolution computed tomographic (CT) scans (see Methods for description) (**Figure 1C**), but not significantly with lung function (**Supplementary Figures 1C, D**). This suggests that monocyte levels are more closely associated with fibrosis, since lung function abnormalities can also be contributed by other factors like cardiac dysfunction, emphysema, and pulmonary hypertension.

There were no abnormalities in frequencies of intermediate (CD14^hi^CD16^hi^), non-classical (CD14^mid^CD16^hi^) and classical (CD14^hi^ CD16^neg/mid^) subsets; nor monocytes with more inflammatory (M1-like - CD62L, CCR7) or pro-repair (M2-like – CD163) features (**Figure 1D** and **Supplementary Figures 1E–G**). However, CD64 expression on monocytes was increased (**Figure 1E**), and was uniformly high in all IPF monocytes. Thus, the total CD64 expression in the monocyte population per patient correlated strongly and positively with CT fibrosis score (**Figure 1F**). CD64 levels were not affected by Pirfednidone and did not correlate with lung function (**Supplementary Figures 1H, I**).

To explore possible causes for, and consequence of, increased monocyte levels and raised monocytic CD64 expression, we examined key inflammatory and monocyte-associated factors in serum from IPF patients collected in the second half of the year (see Methods); (n=24 IPF and n=11 HCs) (demographic data in **Supplementary Table 2**). A set of 13 cytokines and chemokines were selected according to these groups - (i) monocyte activation/differentiation/trafficking factors - CCL-2, CSF-1, TNF-α, IL-6, CXCL-10; (ii) T cell activation/pro or anti-inflammatory/trafficking factors - IL-13, TNF-α, CCL-20, CXCL-9, IFN-γ, IL-6, IL-1β, IL-10, and (iv) granulocyte differentiation/trafficking CSF2, CXCL8.

We found a significant increase in the levels of CSF-1, IL-6, and CCL-2, in IPF serum compared to HCs [793.7(228.5) vs 250.6(102.3) pg/ml, p<0.001;7.0(3.5–25.5) vs 1.6(1.0–3.2) pg/ml, p=0.02; and 1,087.0(916.2–1186.0) vs 650.8 (531.0–770.0) pg/ml, p=0.001, respectively) (**Figures 1G–I**). No other analytes were significantly different from controls (**Supplementary Figures 2A–K**). The outcome of all analyses was the same when patients with Pirfenidone were excluded from the analyses (**Supplementary Figure 2L**). Correlation analyses showed a modest correlation between monocytic CD64 expression and CSF-1 levels only (**Supplementary Figures 2M, N**).

These results showed that IPF patients have increased blood monocyte levels that correlated with extent of lung fibrosis, accompanied by high serum CSF-1, CCL-2, and IL-6 levels. IPF monocytes displayed higher levels of CD64 expression, a high-affinity IgG receptor (FcγRI) and marker of type I interferon activation.

### Bulk RNA Sequencing Supports Type 1 IFN Signature in Monocytes From IPF Patients

To explore if the transcriptome of monocytes supports a type 1 IFN primed phenotype, we isolated monocytes from IPF and age-matched healthy controls for bulk RNA sequencing. Monocytes were isolated using CD14^+^ magnetic bead selection (98%–99% purity) from three well-characterized IPF patients (with definite UIP pattern fibrosis on CT scan) who were not on anti-fibrotics, and were non-smokers (all male; aged 57, 76, and 78y) and three healthy controls (HC) (non-smokers, no medications; aged 65, 68, and 71y; all male).

**Figure 2:**
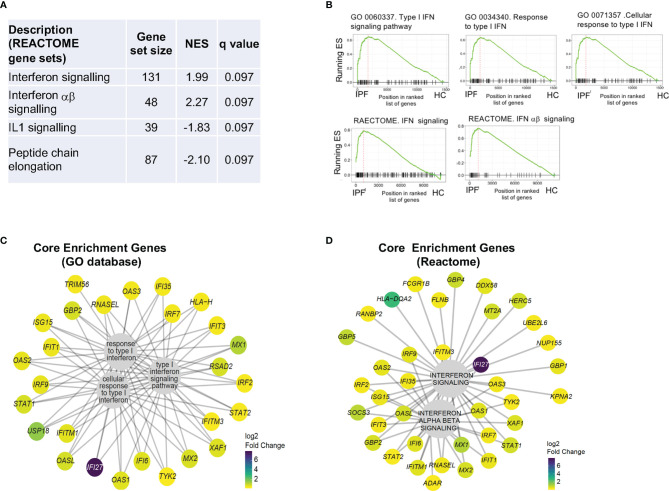
RNA sequencing of IPF monocytes reveals a Type I IFN signaling signature. **(A)** Analysis of differentially expressed gene list for enrichment of biologically-relevant pathway and processes using REACTOME gene sets. NES – normalized enrichment score. **(B)** GSEA plots showing most enriched gene sets from GO and REACTOME database. ES- Enrichment score. **(C, D)** Composition and expression levels of genes in the leading edge of the five gene sets from **(B)** expressed as log2 fold change.

**Figure 3:**
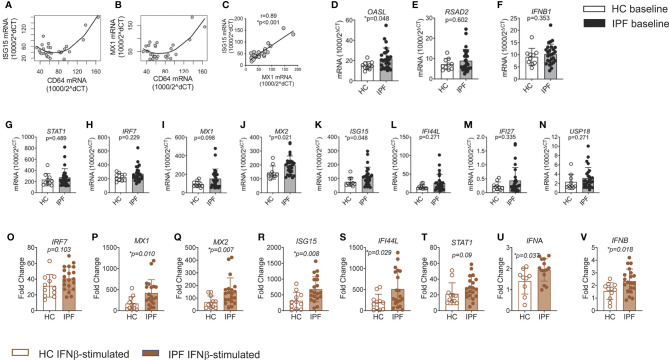
Amplified response to type I IFN stimulation in monocytes from IPF. **(A–C)** Correlation of CD64 mRNA expression levels in IPF monocytes with ISG15 and MX1 levels; and between ISG15 and MX1 **(C)**. **(D–N)** ISGs, IFNB1 and IFNAR expression in freshly isolated IPF monocytes vs HC, using qPCR. Results were expressed as relative gene expression - 1,000/2^(CT of target gene−CT of housekeeping genes). **(O–V)** Increased levels of ISG, *STAT1, IFNA, and IFNB* mRNA expression in IPF monocytes after culture in recombinant type 1 IFN for 18 h. Fold change refers to change over unstimulated values for that patient’s monocytes.

Enrichment analysis of differentially expressed genes between IPF and HC using REACTOME platform and Gene Set Enrichment Analysis (GSEA), showed that the most significantly enriched gene sets were “IFN signaling” and “IFNαβ signaling” (**Figures 2A, B** and **Supplementary Tables 3A, B**). Examining the genes that contributed most to the enrichment of the five IFN signaling gene sets shown in **Figure 3B** (“core enrichment genes”), we observed that most of the genes were interferon stimulated genes (ISGs) from different points in the type 1 IFN signaling pathway (**Figures 2C, D**) (26).

These findings, from an unbiased and whole transcriptome approach, provide support for (but does not establish) the link between increased CD64 expression and type I IFN pathway activation in IPF monocytes.

### Monocytes From IPF Patients Show Greater Response to Type I IFN Stimulation *Ex Vivo*

To explore the possibility that increased CD64 signified an activated type I IFN pathway in IPF monocytes, we first examined contemporaneous basal expression of CD64 *(FCGR1B)* and representative ISGs (*MX1* and *ISG15*). We observed a strong correlation between all three genes (positive between MX1 and ISG15 and curvilinear between CD64 and MX1/ISG15), supporting the link between CD64 expression and type 1 IFN signaling (**Figures 3A–C**). As expected, MX1 expression was tightly linked to ISG15 (**Figure 3C**). Demographic details are found in **Supplementary Table 4**.

We next examined baseline gene expression (qPCR) of archetypal interferon stimulated genes (ISGs) *IFI27, USP18, MX1*, *OASL, IRF7, and MX2*, and those ISGs found highly expressed in PBMCs from the type 1 interferonopathy, Aicardi-Goutières syndrome (AGS) patients (*IFI27, ISG15, IFI44L, and RSAD2)* (27). *IFNB1 and STAT1*, a key transcription factor in the type I IFN signaling pathway were also determined.

All ISGs, *STAT1*, and *IFNB1* were more highly expressed in IPF compared to control monocytes, with some statistically significantly increased - MX2, ISG15 and OASL (p=0.021, p=0.048 and p=0.048 respectively) (**Figures 3D–N**). There was no difference in findings between patients on anti-fibrotics and those not (**Supplementary Figure 3**). Neither *IFNB1* nor *IFNAR1* expression was significantly increased in IPF monocytes at baseline. However, basal ISG expression levels were significantly correlated with basal *IFNB1* and *IFNAR1* expression (**Supplementary Figure 4**). This observation provided support for the integrity of the data as *IFNB1* and *IFNAR1* and ISG genes are expected to change contemporaneously.

Next, we tested if IPF monocytes showed an enhanced response to type 1 IFN stimulation. Freshly isolated monocyte from IPF patients were stimulated with 100U/mL recombinant human IFN-β for 18 h and expression of key ISGs (*STAT1, IRF7, MX1, MX2, ISG15, IFI44L*), *IFNA*, and *IFNB1* were examined. Time point and dose for stimulation were selected due to previous work suggesting that these were the most appropriate time points and levels (28).

Monocytes from IPF patients showed a greater response to type I IFN stimulation for *MX1*, *MX2*; *ISG15* and *IFI44L* (p=0.010, p=0.007, p= 0.008, and p= 0.029 respectively) (**Figures 3O–S**), regardless of the use of anti-fibrotic (**Supplementary Figure 5**). The induction of both *IFNA* (p=0.04) and *IFNB* (p=0.02) was also significantly greater in IPF monocytes (**Figures 3T–V**). These results are in keeping with a primed type I IFN signaling pathway, along the JAK-STAT signaling arm of the pathway (**Supplementary Figure 6**).

These findings show that CD64 expression in individual monocytes correlates with ISG levels, and that IPF monocytes have an activated type I IFN signaling pathway and are primed to respond to type 1 IFN stimulation. These findings support the likelihood that increased CD64 expression in IPF monocyte is linked to type 1 IFN priming.

### Single Cell Transcriptomic Analysis of IPF Lungs Reveals Increased Proportion of Pathological Monocytes and “Transitional Macrophages” Enriched With Type 1 IFN Signatures

In our final study, we questioned if our findings in the blood were also observed in the lungs. To do this, we interrogated the single cell transcriptomic data from Reyfman et al. (10) to specifically determine if myeloid cells in the lungs of IPF patients, compared to healthy controls, displayed increased CD14, CD64, and enrichment of type I IFN gene sets.

**Figure 4:**
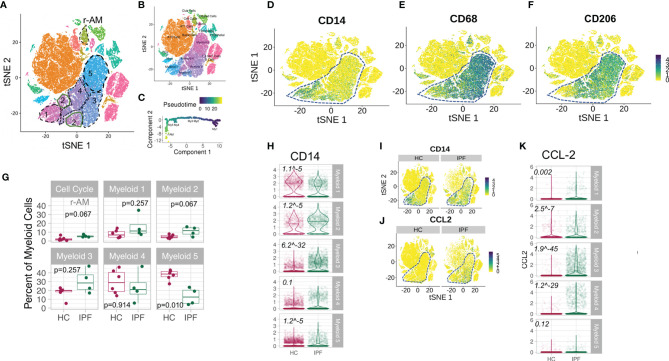
Increased number of CD64^hi^ monocytes and CCL-2-expressing transitional macrophages in IPF lungs. **(A)** tSNE plot of all lung cells from lung digest of n=4 IPF explants and n=6 healthy control lungs. Broken lines outline the five subsets in the myeloid cell cluster. r-AM refers to a separate subset of cells with high cycling RNA content, some expressing CD14, CD68, and CD206 which are likely to represent embryonically-derived resident alveolar macrophages. These were referred to as “Cell Cycle” subcluster in other plots. **(B)** tSNE plot showing annotation of other immune cells as per Reyfman PA et al. (23). **(C)** Pseudotime analyses of transcriptomic states (Monocle) showing the direction of progression of states from My1 to My5. **(D–F)** tSNE plots showing CD14, CD68, and CD206 expression in all populations of cells from lung digest (composite of four IPF and six HC) showing progression of expression in myeloid cluster [(My1-5) in broken outline] in keeping with My1 being monocytes, My2-3, “transitional macrophages” and My4-5 being more mature macrophages. **(G)** Graphs showing subclusters as % of myeloid cluster. P value derived using Wilcoxon rank sum testing. **(H)** Violin plots for CD14 expression on each subcluster (My1-My5). Italic number refers to FDR q values comparing HC and IPF. **(I, J)** tSNE plots for CD14 and CCL-2, expression on all cells from lung digest, showing predominant expression in myeloid subsets (in broken outline) and increased expression in IPF compared to HC in the myeloid subsets. **(K)** Violin plots for CCL-2 expression on each subcluster (My1-My5). Italic number refers to FDR q values comparing HC and IPF.

Reyfman and colleagues performed single cell RNA sequencing using 10X technology on lung tissue obtained from lung transplant donors [“healthy controls” (HC)] and explanted lungs from IPF recipients. We selected 4 IPF patients (Sample ID IPF1, 2, 3, and 4) and n=6 healthy control donor lungs (Sample ID Donor 1, 2, 3, 4, 6, and 8) and excluded Donor 5 and 7 as they were current cigarette smokers. Seurat (with Wilcoxon rank test) was used to identify clusters (21). Batch correction (as described in Methods) revealed that each cluster consisted of cells from each donor following multiple sample integration (**Supplementary Figure 7**). Clusters were annotated based on expression of canonical marker genes, as provided by Reyfman et al. (10). Our annotation revealed 22 clusters which broadly matched Reyfman’s (**Figures 4A, B**). We focused specifically on the myeloid cells, within which were five transcriptomically distinct subclusters, which we termed Myeloid1-Myeloid5 (My1-5). Expression of CD14 (monocyte), CD206 (macrophage), and CD68 (macrophage) on tSNE plots suggests that My1 subcluster were monocytes (CD14^+^CD206^neg/lo^CD69^neg/lo^), My2 were early transitional macrophages (CD14^+^CD206^lo^, CD68^lo^) My3 later transitional macrophages (CD14^+^CD206^lo^, CD68^mid-hi^) and My4 and 5 were macrophages (CD206^mid-hi^CD68^mid-hi^ CD14^neg/lo^) (**Figures 4D–F**). This was supported by pseudotime analysis of the myeloid cluster using Monocle pseudotime (29), which constructs a single cell trajectory of transition from one state to another. Here, a dynamic repertoire of transcriptomic changes suggests a progression in transcriptomic states from My1 to My5 (**Figure 4C**) (we instructed the algorithm to use My1 as the starting point due to clear presence of CD14 expressing cells in this subcluster; **Figure 4D**). In the tSNE clustering, we also noted a population of cells (“r-AM”; **Figure 4A**) with high levels of cell cycling genes, consistent with self-renewal or proliferation. This cluster did not contain epithelial markers but contained a large proportion of CD206 and CD68 expressing cells, which suggested that they are the embryonically-derived, self-renewing resident alveolar macrophages.

Having annotated the myeloid population and its subclusters, we examined the differences in these clusters between IPF and HC. We observed a greater proportion of monocyte and transitional macrophages (My1, 2, and 3) in the myeloid population in IPF lungs, with significantly lower proportion of mature macrophages (My4 and My5) (**Figure 4G**). The proportion of mature macrophages was correspondingly reduced (**Figure 4G**).

CD14 expression was higher in all sub clusters of myeloid cells in IPF lungs, except the earliest monocyte cluster (My1) (**Figure 4H**). This increase in CD14 was most pronounced in the transitional macrophages (My2 and My3) (**Figures 4H, I**). In view of our findings in the serum, we also questioned whether the high serum CSF-1, CCL-2, and IL-6 could have originated from cellular sources in the lungs. Interrogation of the entire cell population (i.e., not only the myeloid cluster) showed no evidence of increased IL-6 expression in any immune or structural cells in IPF (**Supplementary Figure 8**). However, CCL-2 expression was significantly increased in My1 to My4, most pronounced in the late transitional macrophages, My3 (**Figures 4J, K**). These findings suggest that high CCL-2 in the serum may be secondary to high CCL-2 levels in the lungs which may provide the chemoattraction for monocytes to the lungs (although this is not established here). We did not observe significant increases in IL-6 of CSF-1 expression in myeloid cells from IPF compared to HC (**Supplementary Figure 7**).

**Figure 5:**
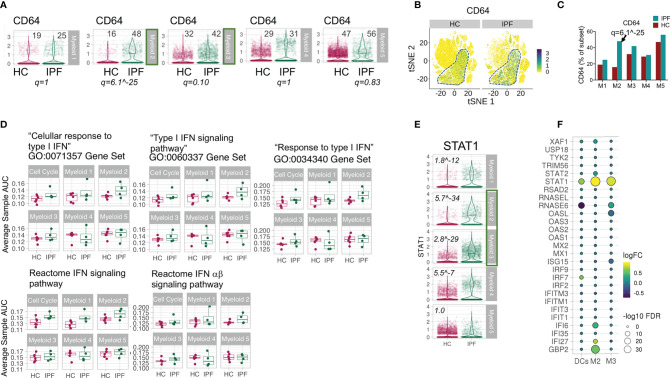
Increased markers of type I IFN pathway in myeloid cells in IPF lungs. **(A–C)** Violin plots, tSNE and graph showing expression of CD64 in My1-5 subsets in lung digest. Numbers in plot of **(A)** refers to % of CD64 expressing cells in myeloid subcluster for HC and IPF patients. FDR q values applies to difference in expression between HC and IPF. **(D)** Average cell AUC value per sample per cluster representing enrichment of gene set in the transcriptome comparing IPF and HC samples. All p values >0.05 except for Myeloid 1 (My1) in Reactome IFN signaling pathway gene set - p=0.04 (Wilcoxon Rank Sum Test). **(E)** STAT1 expression in My1-5 depicted as violin plots. Italic values are FDR q values comparing HC and IPF. **(F)** Bubble plot reflecting expression of ISGs in My2, My3, and dendritic cells (DCs) depicted as fold change and FDR q, comparing IPF and HC, showing increased ISGs in My2 and My3 but not in DCs.

In our final analyses, we examined if the myeloid cells in IPF lungs also showed a type I IFN signature. CD64 expression was increased in IPF lung myeloid cells across My2-5 myeloid subclusters, most significantly higher in My2 and 3 (FDR q = 6.12^-25^ and 0.1 respectively) (**Figures 5A–C**). There was also enrichment of all type I IFN gene sets in My1-3 clusters in IPF compared to HC, in contrast to My4-5 subclusters (**Figure 5D**). Analyses of leading ISGs showed clear increase in STAT1 expression in My1 to 4 (**Figure 5E**), most pronounced in the transitional macrophage clusters (My2-3) (**Figure 5F**). As a comparator, IPF’s dendritic cells did not show the raised ISG levels observed in the transitional macrophages (**Figure 5F**).

To explore how our annotations and findings compare to published data set, we obtained data from Adam et al. (23) which were selected as they provided data that have publicly available annotations. The comparison of the myeloid cell cluster showed broadly aligned subclusters of myeloid cells between the two (**Supplementary Figure 8**). My1 were analogous to Adam’s non-classical and classical monocyte populations, and My2-5 to their inflammatory and fibrotic macrophage clusters (**Supplementary Figure 9**). Interestingly, when My2-5 were interrogated for the defining fibrotic and inflammatory genes derived from Adam’s inflammatory and fibrotic macrophage clusters, My3 showed closest alignment to the fibrotic macrophage clusters in Adam’s dataset (**Supplementary Figure 10**). My3 was the transitional macrophage with higher CD64 and type I IFN gene enrichment amongst our myeloid sub-clusters. These observations suggest that the data are robust, and raise the possibility that My3 subcluster has pro-fibrotic function.

Overall, this high definition data in the lungs show that there was a greater proportion of monocytes and transitional macrophages (My1-3), with a reduction in mature macrophage subsets within the myeloid cell population. The My2-3 (transitional macrophages) subclusters in IPF showed increased levels of CD64, STAT1, CD14, and enrichment of gene sets compared to HC. My3 may be analogous to the fibrotic macrophage subcluster proposed by an independent dataset (23). These subclusters and the My4 subsets also showed significantly higher levels of CCL-2 expression in IPF compared to healthy controls.

## Discussion

Our results show for the first time, that circulating monocytes in IPF are endowed with a primed type I IFN pathway, likely marked by increased CD64 expression. In the lungs, high definition single cell transcriptomic studies revealed corollary increase in specific clusters of myeloid cells with transitional monocyte-macrophage features, also marked by increased levels of CD64, CD14, and CCL-2 gene expression; and enriched type I IFN signaling gene sets. A striking finding of increased CSF-1 levels in the serum was also observed.

Enhanced responsiveness to type 1 IFN in monocytes is a highly significant finding for IPF patients. Type 1 IFN signaling is activated by two consecutive pathways. The pathway can be triggered by sensing of self-nucleic acid or viral nucleic acid and bacterial pattern-recognition molecular patterns (PAMPS) (30). In both these scenario, nucleic acids are recognised by surface TLR4, endosomal TLR3, 7, 8, 9 or other cytosolic sensors (**Supplementary Figure S6**) and results in transcription of some ISGs and type 1 IFNs by the cell. This type 1 IFN then ligates the IFN receptor (IFNAR) on the same or neighbouring cells and cause transcription of hundreds more ISGs which directly interfere with pathogen replication and also activates other immune pathways. This potent and early immune defence pathway unleashes a large number of cytokines, chemokines (31, 32), and also enhances natural killer cell function and high affinity T and B cell responses (28). In non-infectious settings, when Type 1 IFN signaling is persistently activated [e.g., due to the recognition of self-nucleic acid in systemic lupus erythematosus or abnormal nucleic acid species in Aicardia-Goutieres syndrome (AGS)] severe inflammation and clinicopathologic manifestations can ensue (27).

One possible consequence of having a primed type I IFN pathway is a magnified downstream innate and adaptive immune response in IPF patients when they are infected. This could cause widespread alveolar injury as observed in acute exacerbation of IPF. Type I IFN can also promote epithelial senescence (33), a key factor in IPF pathogenesis by amplifying DNA-damage responses and activate the p53 pathway. However, another way of looking at this is that a primed type I IFN state in monocytes is advantageous. Thus, viral infections could be dealt with much quicker. Intriguing murine studies have also emerged which showed that IFN-β secretion by satiated macrophages can promote neutrophil apoptosis and efferocytosis and differentiate macrophages towards an inflammation-resolving phenotype (34, 35). The key question is probably whether monocytes could switch off the type I IFN response as rapidly as it is deployed. Further studies to elucidate the consequence of activation of these primed monocytes would help decipher the pathogenic role of these monocytes further.

Another important question is the cause of type I IFN signaling priming. Type I IFN signatures have been found in immune cells in SLE, thought to be a response to self-nucleic acids and their associated nuclear proteins. Apoptotic and necrotic cells as well as neutrophils undergoing a specific form of cell death called NETosis could also be sources of these self-nucleic acid. Accordingly, these are possibilities in IPF since injured alveolar epithelium can be a source of these damage-associated molecular patterns (DAMPS) (36). One other possibility could be the presence of infection in these patients, either acutely or latent. In terms of the former, this is unlikely as all patients were carefully vetted for clinical infections by at least two physicians (at the point of sampling, and also in clinic on the same day). We did not observe high levels of IFN-α or β in the serum to account for presence of acute infection or indeed a driver for the primed pathway. However, IPF patients have been shown to have greater levels of latent viral particles, e.g., Herpes and Epstein Barr viruses in lung biopsies (37–39). Although these viruses tend to be kept in check by T cells, there is evidence that type I IFN pathway may also be involved (40), so this could potentially be a cause for the primed type I IFN signaling in these patients. Finally, an epigenetic influence, possibly from long term “training” of innate immunity, on the transcription of type I IFN signaling could also prime the type I interferon pathway in monocytes (4). The interferon regulatory factors (IRFs) are under epigenetic control (41) and can be modulated epigenetically to increase type I IFN signaling. These possibilities will be interesting to explore in future studies.

Presence of greater proportion of transitional macrophages (potentially, immature functionally) with monocytic features in the lungs is also a very significant finding from our study. This is in keeping with an important finding from Aran and colleagues in mice (42) who showed that deletion of a transitional macrophage cluster in murine lungs after bleomycin challenge prevented lung fibrosis. In our analysis, the immature/transitional macrophage clusters also expressed higher levels of the monocyte chemoattractant, CCL-2 in IPF, potentially attracting more abnormal monocytes to the lungs. A recent study in IPF lungs also showed reduced phagocytosis in macrophages in the lungs which could reflect the immature state of macrophages (43). This could have pro-fibrotic implications since macrophages are important effector cells in mediating repair and limiting inflammation after the initial stages of injury. They scavenge cellular debris, dying neutrophils and other apoptotic cells, and release IL-10 and other factors that regulate and control extracellular matrix deposition (44). Therefore reduction of the more mature macrophages may reduce these anti-fibrotic functions in the lungs.

A primed type 1 IFN state could also reflect a satiated post-phagocytic state (34, 35). Interestingly, this satiated macrophage state appears to be associated with increased IFN-β although not IFN-α production (33, 34). A recent study suggests that these IFN-β producing satiated macrophages are associated with an anti- fibrotic phenotype (45). This calls into possibility the differential contribution of the type 1 IFN subtypes to fibrosis and further studies in this area.

It is noteworthy that even at a late stage in disease (explanted lungs), monocytes and less mature macrophages continue to be present, supporting a sustained role for monocytes in advanced disease.

The high CSF-1 levels were a striking finding. This could mean that immature monocytes in the bone marrow were conditioned to differentiate quicker to mature monocytes, providing a potential source of increased monocytes in the blood of these patients (46). Notably, CSF-1 was not found to be expressed by monocytes or macrophages in the lungs, nor the small amount of epithelial cells observed in our single cell transcriptomic analyses. The source of CSF-1 therefore remains unclear but it is known that CSF-1 is secreted by osteoclasts and osteoblasts in the bone marrow, where monocytes are generated and matured (47).

Although our observations are intriguing, it is limited by the relatively small number of patients. We mitigated this by careful selection of patients, ensuring as little confounding factors as possible, and examining both patients taking anti-fibrotics and those not. It is interesting that these monocytic abnormalities are not impacted by anti-fibrotics as it raises the possibility that targeting the monocytic pathway could complement effects of current anti-fibrotics. We also complemented lower number of patients with high definition studies, using cutting edge technologies like single cell RNA sequencing and advanced bioinformatic methods to precisely and accurately determine the clustering and gene expression. Our study is also restricted by being predominantly descriptive which prevents firmer conclusions on how type I IFN-primed monocytes contribute to pathogenesis. However, the un-biased finding of increased CD64 expression, known to be linked to type I IFN activation (48, 49) and more severe prognosis in SLE (48); increased expression of ISGs in IPF blood monocytes and subsequently also in lung monocytes in IPF supports the strength of this observation.

Finally, increased CD64 expression on monocytes of IPF patients may have clinical implications. Proportion of CD64^hi^ monocytes could be a biomarker for patients at risk of a more rapid progression in fibrosis as shown by the correlation with amount of fibrosis in our study, and also to survival by Scott et al. (3). In a small longitudinal analysis of the COMET cohort, Moore and colleagues also showed that increased levels monocytes could be correlated to progression in disease (50).

Our data showed an unexpected finding of type I IFN-primed monocytes in IPF, which could contribute to pathogenesis of IPF. These findings provide an impetus to further examine the contribution of monocytic pathway to pathogenesis of IPF, potentially as a driver of chronic fibrosis, and as a new therapeutic target.

## Data Availability Statement

The datasets presented in this study can be found in online repositories. The names of the repository/repositories and accession number(s) can be found in the article/**Supplementary Material**.

## Ethics Statement

The study had ethical approval from the local and UK national ethics committee (14/SC/1060 from the Health Research Authority and South Central National Research Ethics Service). The patients/participants provided their written informed consent to participate in this study.

## Author Contributions

EF performed the experiments on monocyte phenotyping, and recruited nearly all the patients. LD performed the type I IFN studies and organized recruitment of patients. KB contributed intellectually to the analysis. CV organized patient samples for and performed type I IFN studies. AA and ER performed bioinformatic analysis on the RNA sequencing data and contributed intellectually to the analysis and to statistical analyses of all the data. AA performed all the interrogation of Reyfman’s data. VI provided statistical analysis and overview of the data and statistical handling of the serum profiling output. ST provided statistical overview of the study. NA performed the chemistry studies for the bulk RNA sequencing. VS and RB were specialist thoracic radiologists who analyzed the high-resolution CT scans for the patients and quantified the extent of fibrosis. RH assessed patient suitability and contributed to recruitment of patients. CC contributed to acquisition and analysis of lung samples. CH performed the Luminex studies and contributed to analysis. NM, RR, and JR contributed intellectually and to the design and methodology of the type I IFN studies. JMR, AS, and YZ contributed to the intellectual discussion of the studies. L-PH conceived and led the analysis for all aspects of the study, supervised all experiments and analysis, and drew together the results and manuscript. All authors contributed to the analysis of their respective part of their studies and reviewed the entire manuscript and its conclusions. EF and LD contributed substantially to the writing of the manuscript. All authors contributed to the article and approved the submitted version.

## Funding

The research was funded by the National Institute for Health Research (NIHR) Oxford Biomedical Research Centre (BRC), Medical Research Council UK (MC_UU_00008/1) and Oxford-UCB Alliance research grant. Training, support, and use of the Visiopharm software platform were supported by the Oxford NIHR Biomedical Research Centre (Molecular Diagnostics Theme/Experimental Pathology sub-theme) Cancer Research UK (CR-UK) grant number C5255/A18085, through the Cancer Research UK Oxford Centre and the Pathological Society of Great Britain and Ireland.

## Conflict of Interest

The authors declare that the research was conducted in the absence of any commercial or financial relationships that could be construed as a potential conflict of interest.
