# Supplementary material for: Multi-Modal Characterization of Monocytes in Idiopathic Pulmonary Fibrosis Reveals a Primed Type I Interferon Immune Phenotype
Source: Front Immunol. 2021 Mar 5;12:623430. doi: 10.3389/fimmu.2021.623430 (PMC7973086; doi:10.3389/fimmu.2021.623430)
Supplement: Supplementary file 1 [file Presentation_1.zip › Supp. Figures 1-10_Supp Tables 1,2,4.PDF]

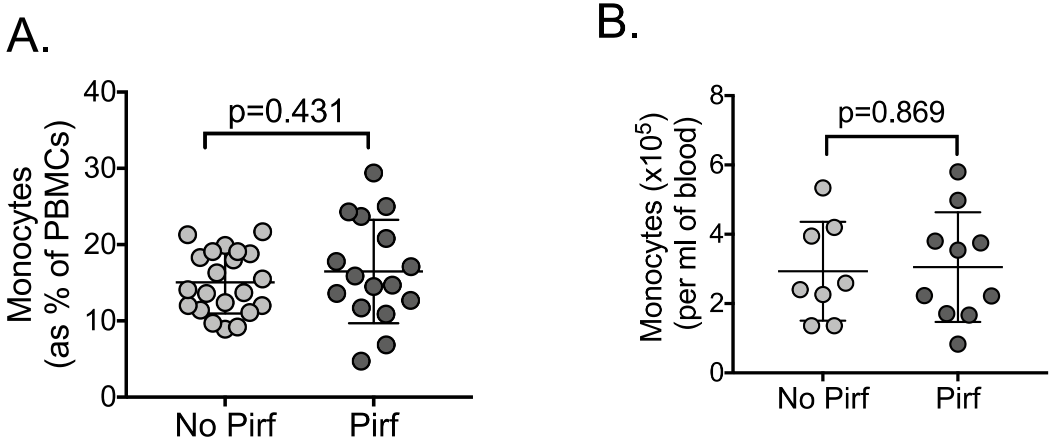

Supplementary Fig. 1A-B Monocytes levels in patients on Pirfenidone compared to without.

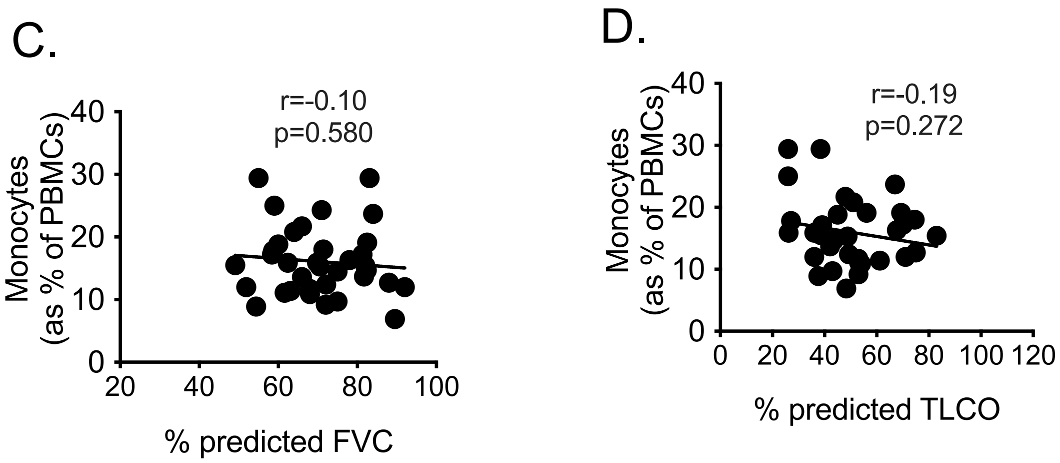

Supplementary Fig. C-D Monocytes levels compared to lung function

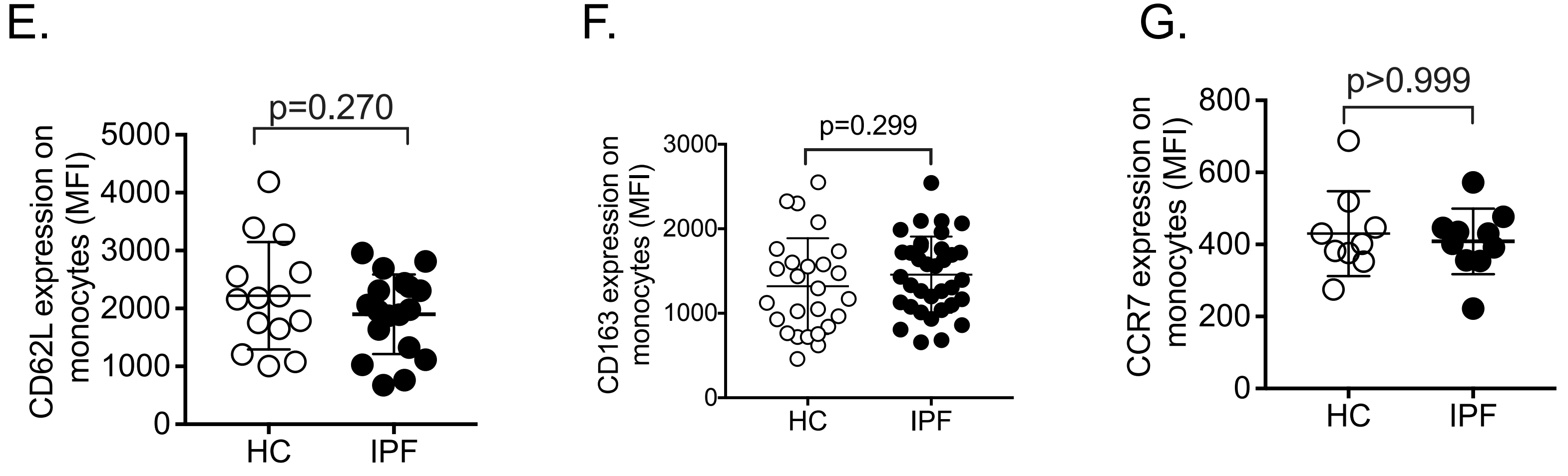

Supplementary Fig. 1E-G Expression of CCR7, CD62L and CD163 on monocytes. MFI – mean fluorescence index.

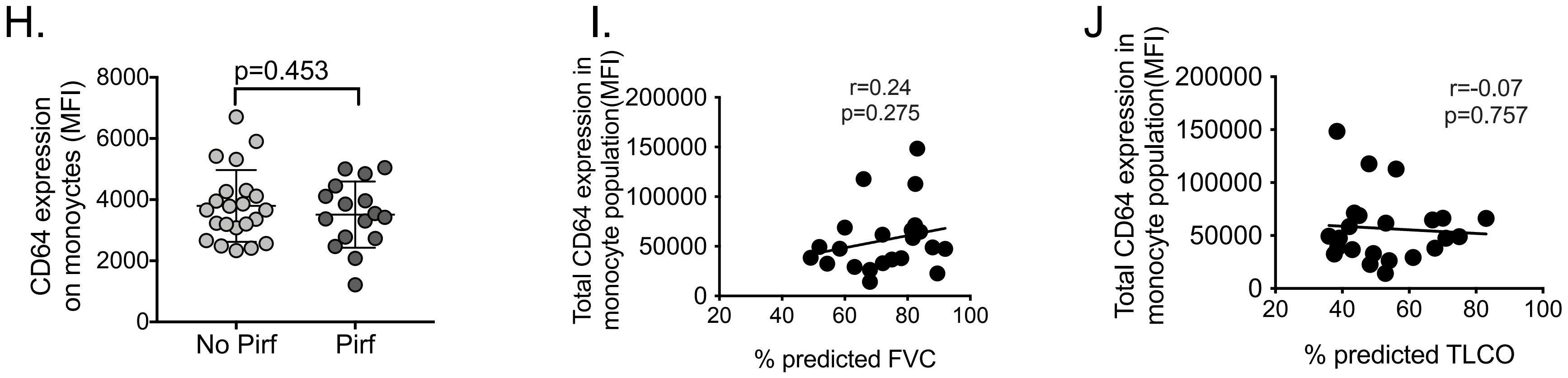

Supplementary Fig. 1H Monocyte expression of CD64 in IPF patients on Pirfenidone (Pirf) and those not on the drug. I-J. CD64 expression on all monocytes per patient vs lung function.

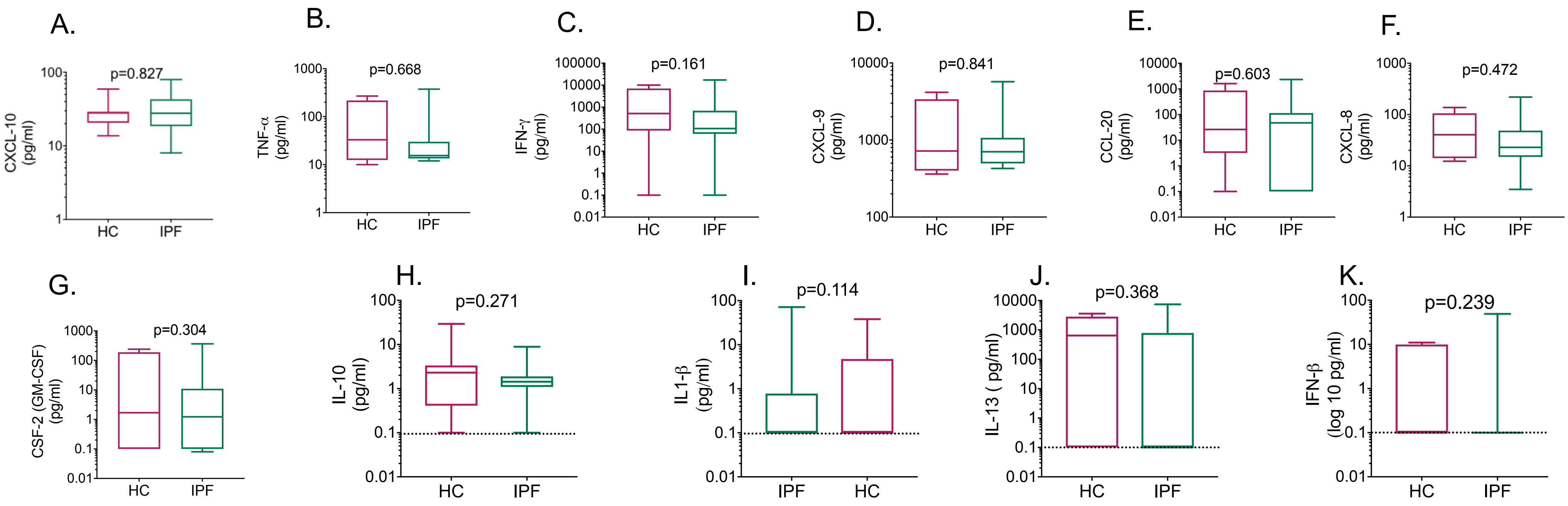

Supplementary Fig 2. A-K Serum analytes' levels (excluding CCL-2, CSF-1 and IL-6 shown in Figure 2) for all IPF vs HC's serum. Serum levels for IL-13, IL-10, IL-1β and IFN-β in both IPF and healthy controls (HC) were not detectable in the majority of patients. Y axis is log 10.

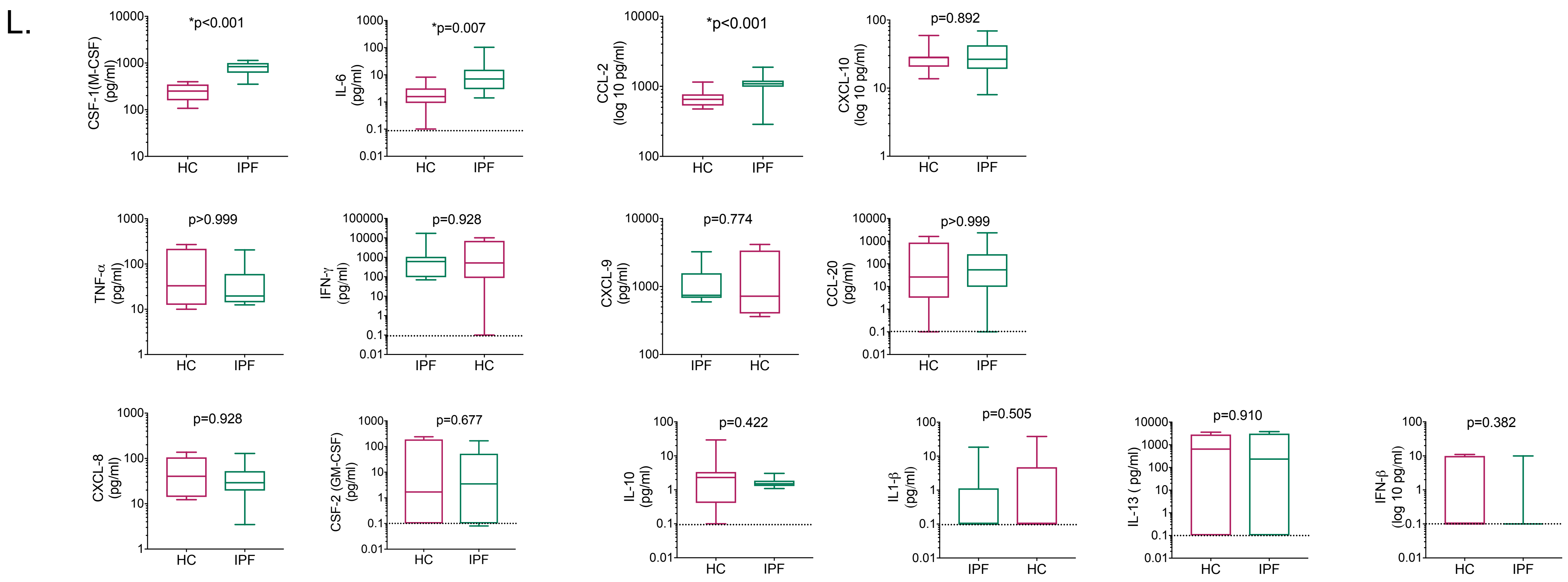

Supplementary Fig 2L. Serum profile of IPF patients – mediators measured in serum as in Figure 2 but without patients on treatment (Pirfenidone or NIntedanib). Y axis is log 10. Zero values for serum mediators converted to 0.1 for visualisation purposes on a log 10 axis. Box plot is mean and S.D, whiskers reflect minimum and maximum values. P values calculated using Mann Whitney Rank Sum testing.

M

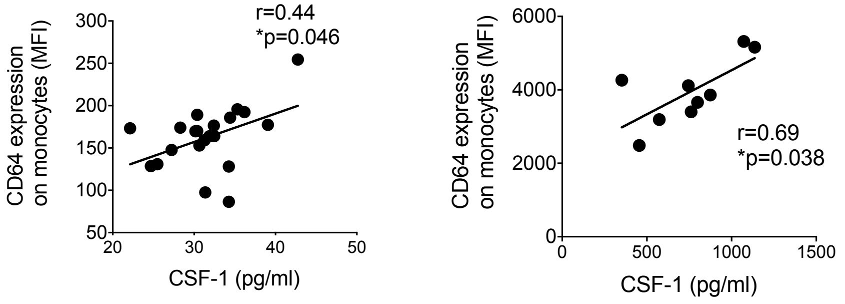

N.

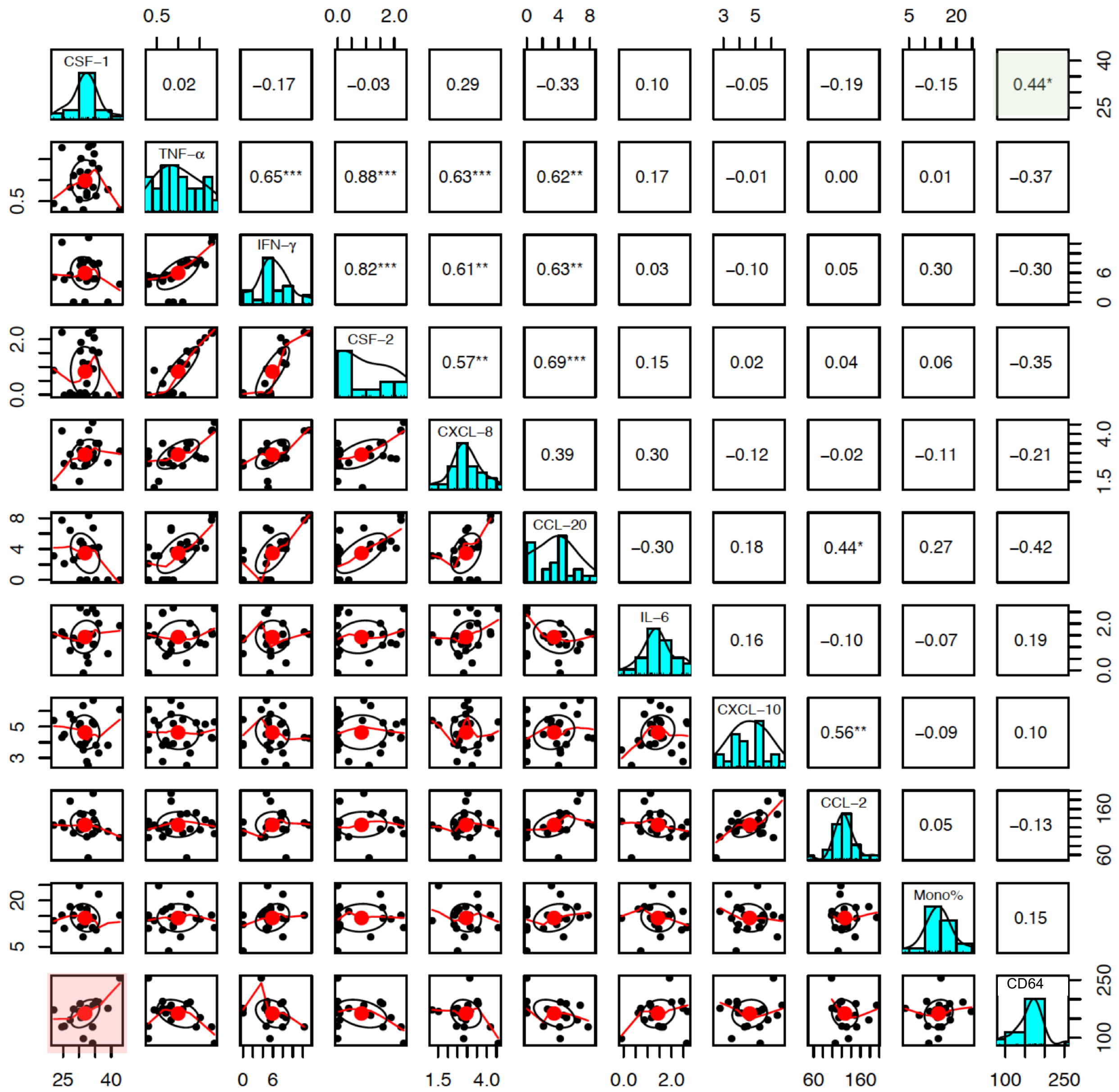

Supplementary Fig 2M-N. (M) Correlation between serum CSF-1 levels and CD64 expression on monocytes (Pearson correlation); for all patients (left panel) and for those not on anti-fibrotics. (N) Pairs plot showing correlation between levels of all soluble mediators, monocyte levels and CD64 expression levels on monocytes. Values in boxes in right hand triangle refers to r value; graphs in boxes in left hand triangle displays scatter plots for corresponding correlation e.g. red box refers to correlation between CSF-1 and monocyte CD64 expression levels; r value is 0. 44 (green box) and it was significantly correlated - **p<0.001; *p<0.05. Bar histogram shows distribution of values for the named parameter. Only values from IPF patients were analyzed. Amongst the soluble mediators, there was a strong positive correlation between mediators that might be secreted by the same cells eg CCL-2 and CXCL-10 from activated monocytes, and IFN-g , TNF-a, CXCL-8, and CCL-20 which may be secreted by lymphocytes. None of these showed different levels comparing IPF and healthy controls. Pearson correlation used for analysis after Box-Cox transformation [$(y^a-1)/a$]. With regards to non-normality of some variables, quantile normalizing the data did not change the conclusions.

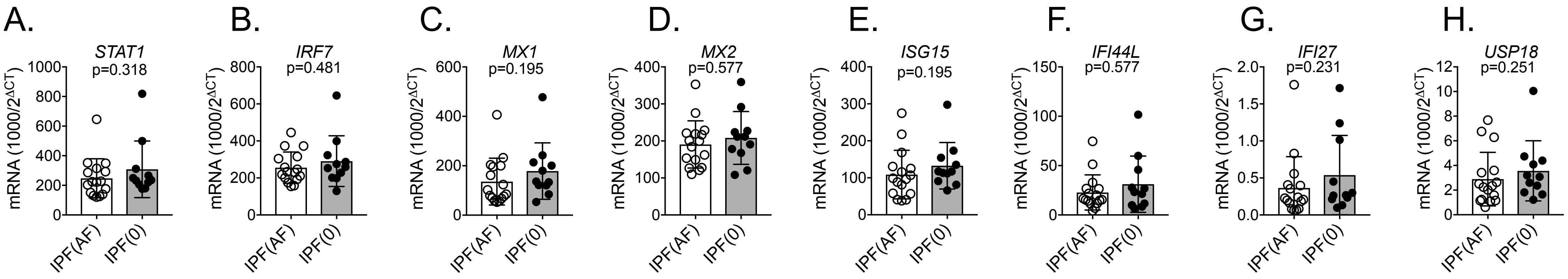

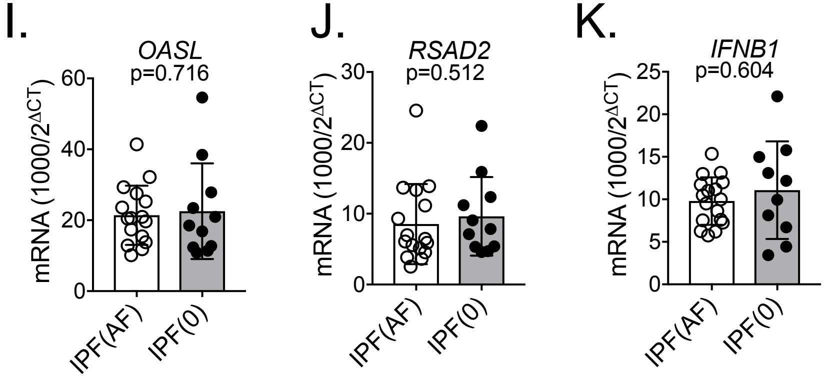

Supplementary Fig 3. Interferon stimulated genes (ISG) expression at baseline (comparing IPF patients who are on antifibrotics (Nintedanib or Pirfenidone) ('AF') and those not on antifibrotics '0'.

L.

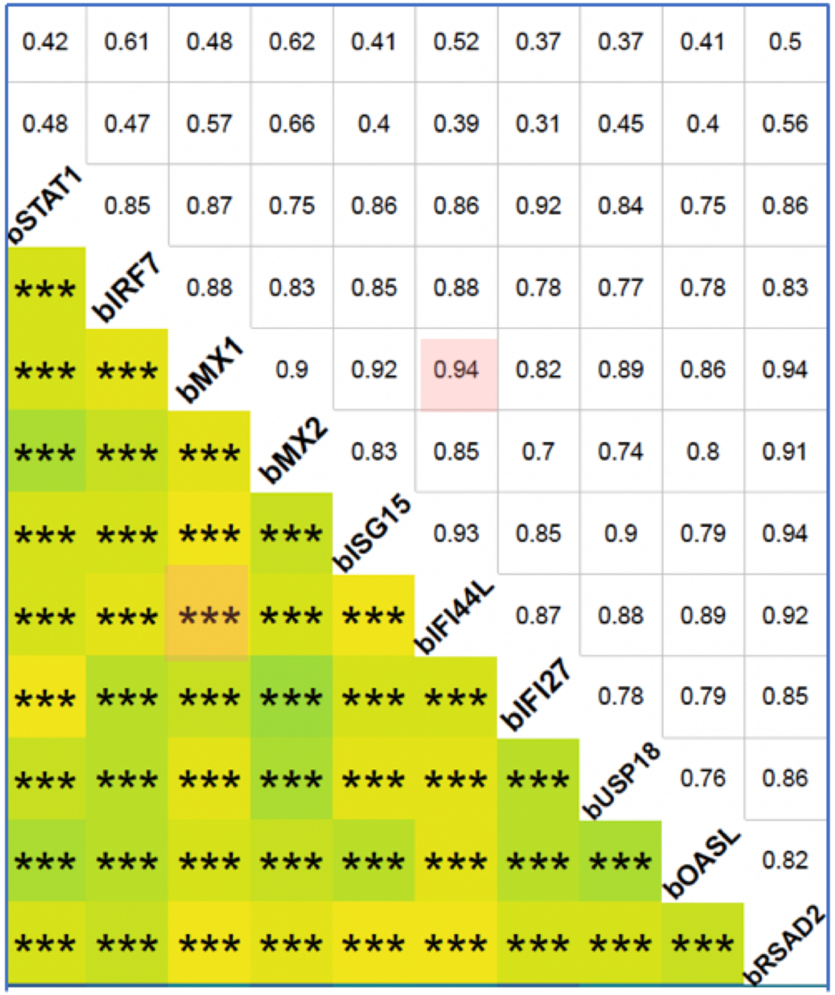

Supplementary Fig 4 Correlation matrix for ISGs, at baseline/unstimulated within an hour of sampling. Numerical values refer to r value (Pearson's correlation) and ** to p value. Color intensity related to strength pf correlation between the two parameters eg basal MX1 expression is strongly correlation with basal IFI44L expression in freshly isolated monocytes from IPF patients; r=0.94 and p<0.001 . 'b' refers to basal gene expression by qPCR

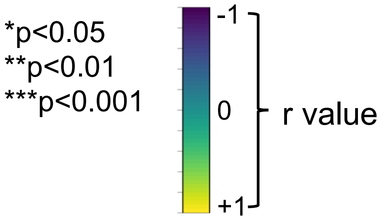

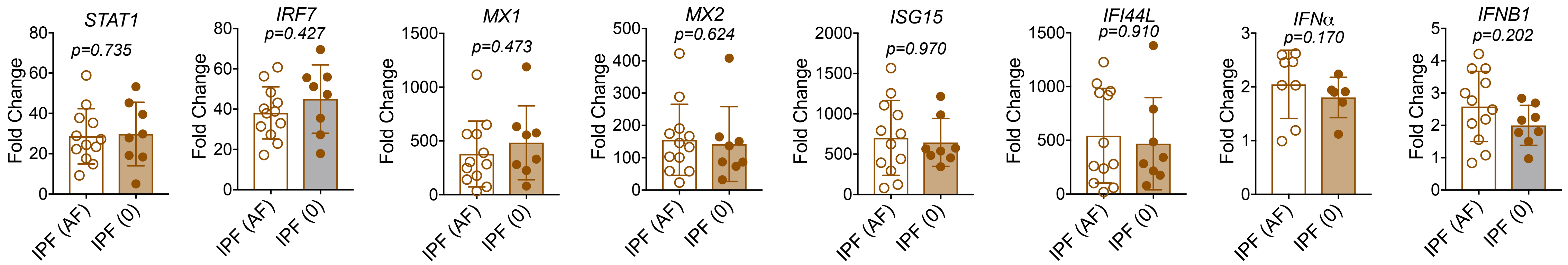

Supplementary Fig 5. Interferon stimulated genes (ISG) after culture in recombinant type 1 IFN for 18 hours ((L-S), comparing IPF patients who are on antifibrotics (Nintedanib or Pirfenidone) ('AF') and those not on antifibrotics '0'. Fold change refers to change over unstimulated values for that patient's monocytes.

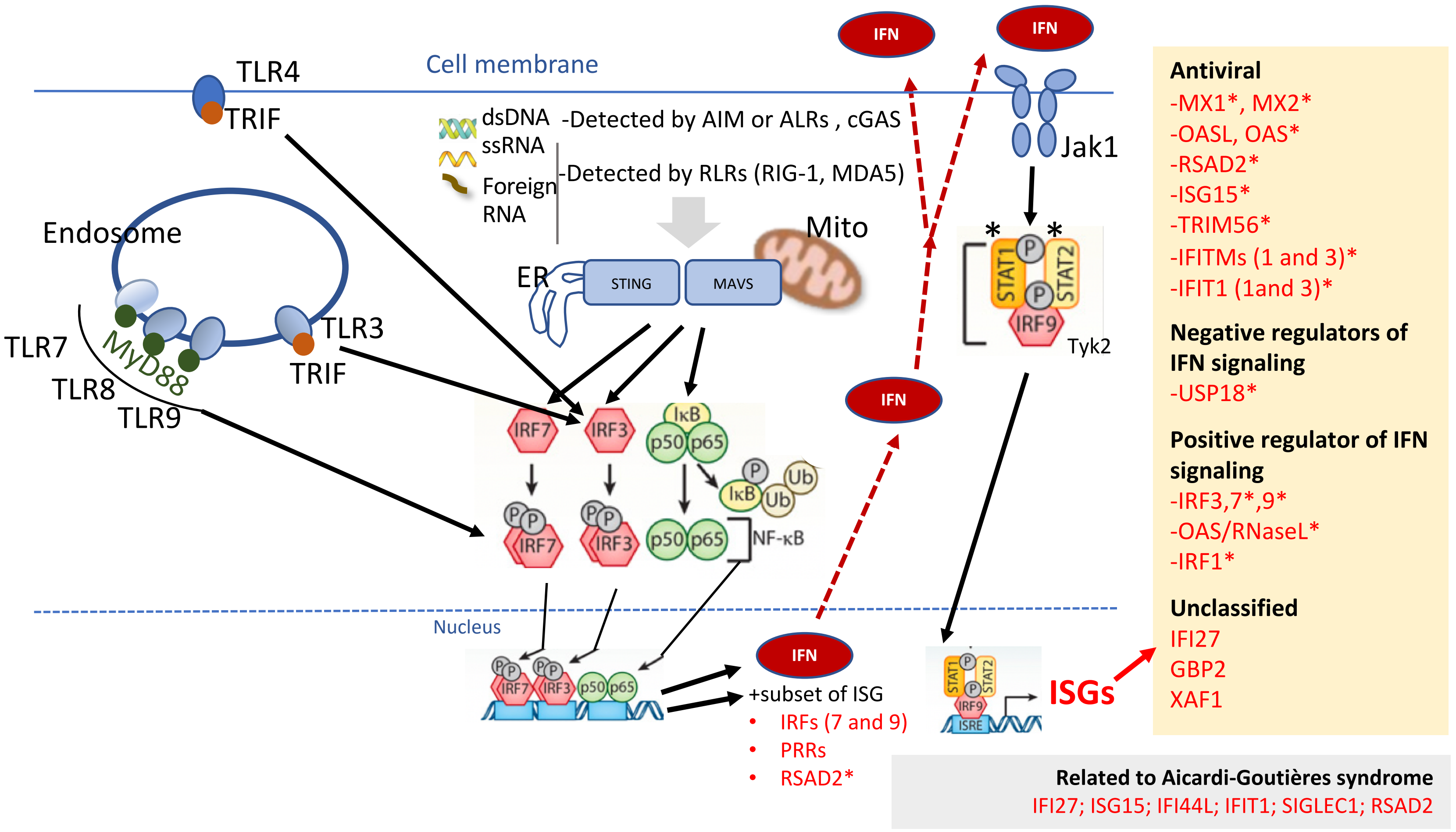

Supplementary Fig 6. Abbreviated type I IFN signalling. The endosomally expressed TLR-3, -7, -8, and -9, cell surface expressed TLR4, the RLRs [retinoic acid-inducible gene I (RIG)-I and MDA-5], and cGAMP synthase (cGAS) can couple pathogen (viral and bacterial PAMPs) and endogenous DNA (PAMPs and DAMPs) detection to type I IFN induction. TLR3 and TLR4 signal *via* TRIF, which occurs through inhibitor of kappa-B (IκB) kinases (IKKs), tumour necrosis factor (TNF) receptor-associated factor (TRAF) family associated NF-κB activator (TANK)-binding kinase-1 (TBK1), and IKK-ε (not shown in figure). This causes the activation of IRF3, which in turn induces the expression of type I IFNs. The activation of TLR3 can also induce the production of inflammatory mediators. TLR7, 8, and 9 use MyD88 for downstream signalling and can activate IRF and NF-κB pathways. RIG-I and MDA5 signal through the adaptor molecule mitochondrial antiviral signalling protein (MAVS). cGAS signals *via* the adaptor protein stimulator of interferon genes (STING). MAVS and STING further recruit signalling molecules (involving the IKK complex, TBK1, and several TRAF proteins) and lead to the activation of NF-κB and IRF3, resulting in gene expression of various antiviral cytokines including type I IFNs. Asterisks denote genes found in leading edge genes from GSEA analysis of monocyte RNA sequencing data (Fig. 3B-D).

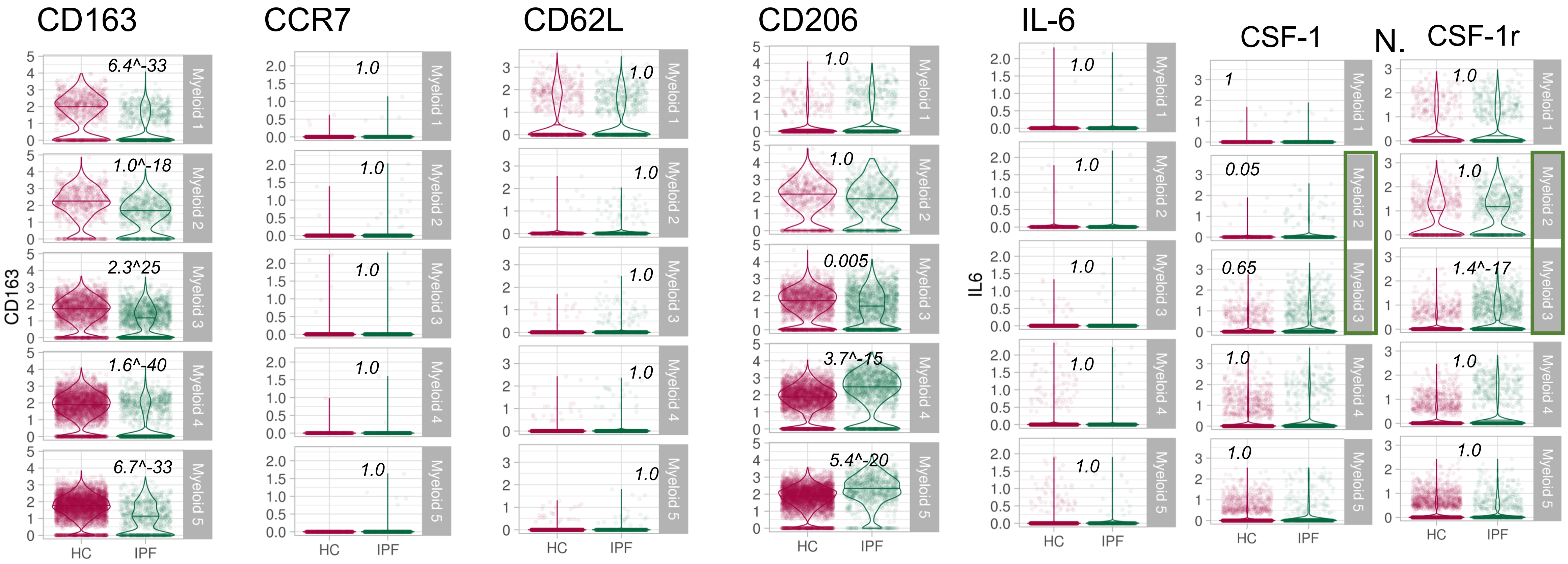

Supplementary Fig 7. Violin plots for expression of CD163, CCR7, CD62L. CD206, IL-6, CSF-1 and CSF-1r comparing HC and IPF for each M1-M5 subclusters.

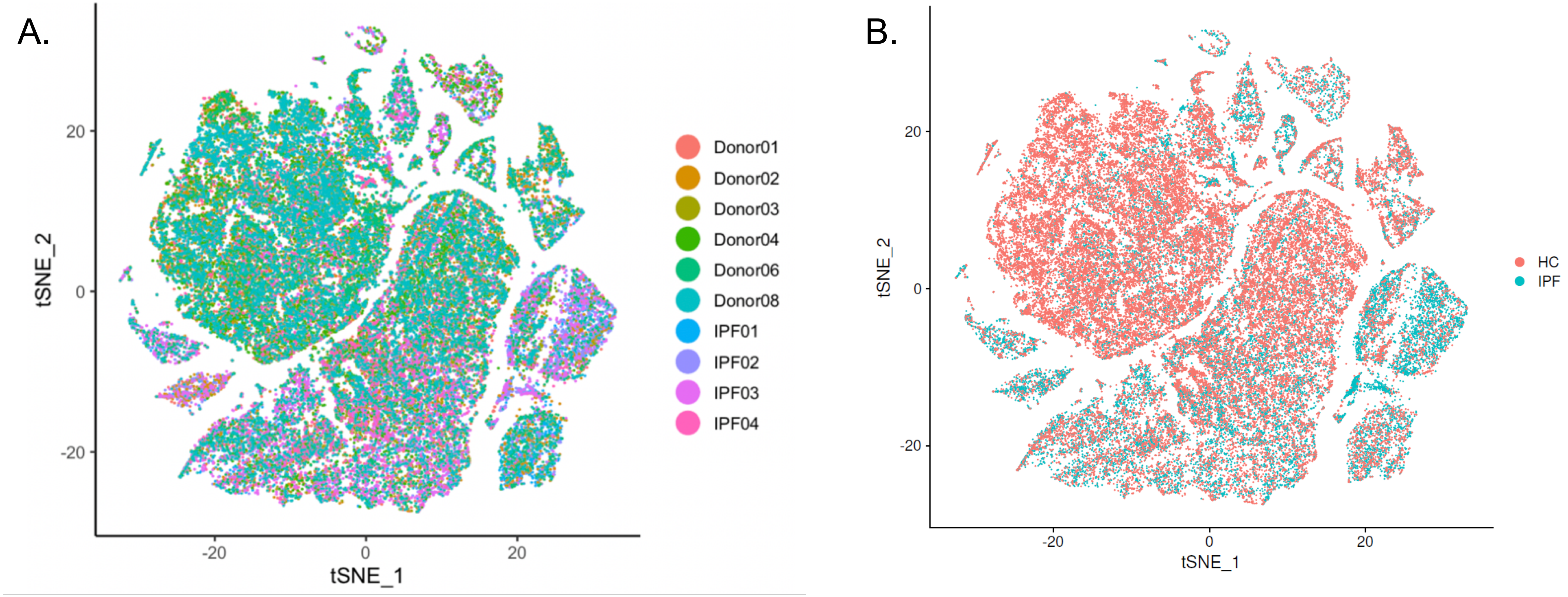

Supplementary Fig 8. tSNE plots of Reyfman's data with each patient (IPF01-04) and control (donor 01-04,06 and 08)'s contribution to the clusters identified by colour (A) and contribution by disease and control also identified by colour in (B). This showed random distribution of cells from each donor, without subclusters within within myeloid clusters, indicating that batch correction has worked.

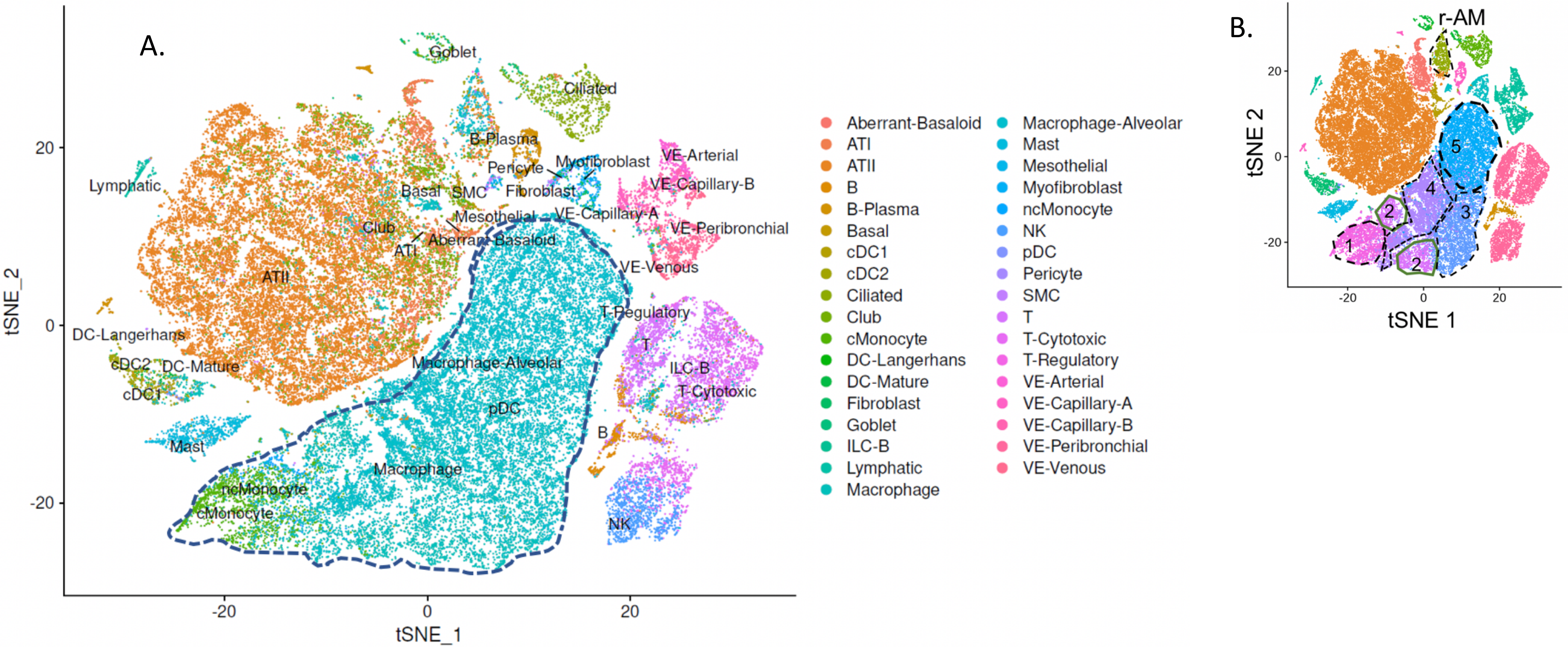

Supplementary Fig 9. A. tSNE plots Reyfman's data clusters, overlaid with annotations from Adam's paper. B. Our annotation in the myeloid clusters for comparison.

A.

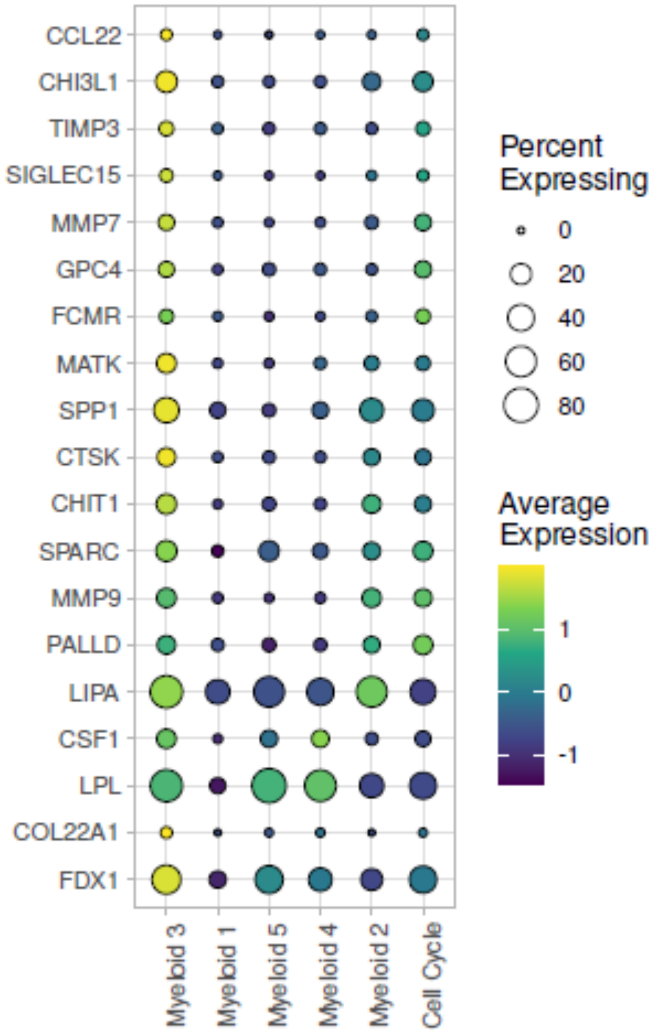

Fibrotic genes
from Adam's fibrotic
macrophages

B.

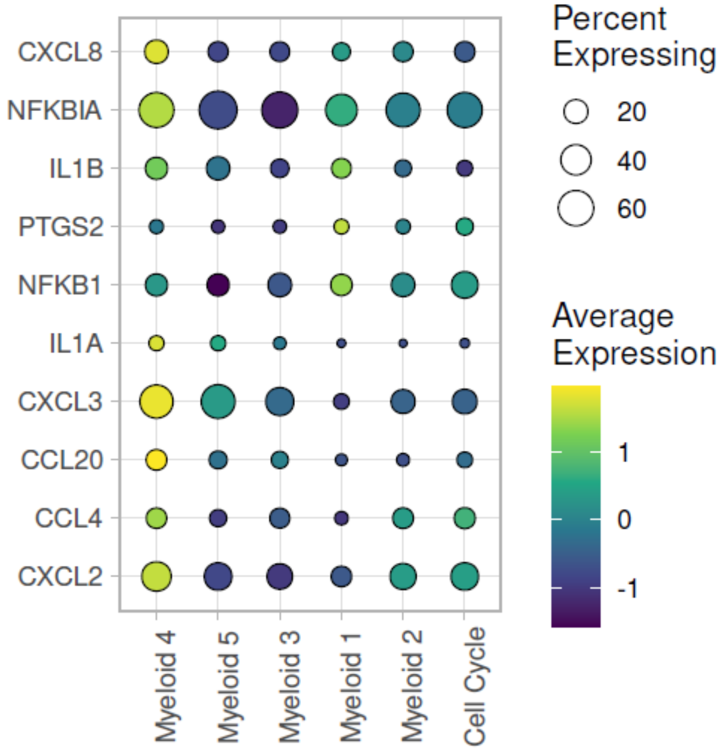

Inflammatory genes
from Adam's inflammatory
macrophages

C.

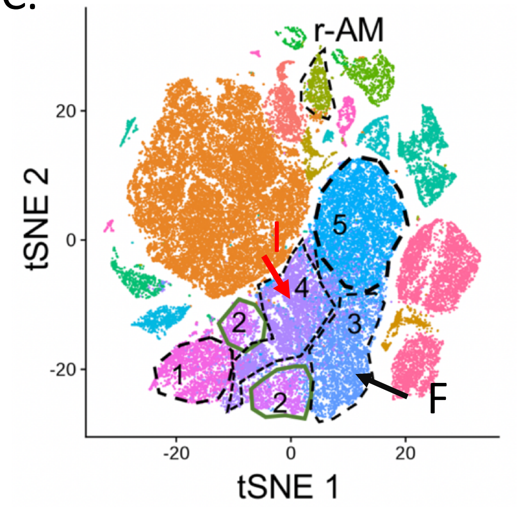

I – putative inflammatory macrophage
F – putative fibrotic macrophage

Supplementary Fig 10. A. Bubble plot for expression and % cells expression of genes of interest – fibrotic from Adam's (A) and inflammatory from Adam's (B). t M4 has the most expression of the inflammatory signature, while M3 has the most expression of fibrotic signature genes. (C).Our annotation in the myeloid clusters for comparison.

IPF cohort	IPF	Controls
n	28<br>(37 samples)	28<br>(28 samples)
Male	23	19
Mean age (range)	72.9y<br>(57-87)	66y<br>(44-81)
On Pirfenidone	14	N/A
Mean FVC<br>(range)	71<br>(48-124)	N/A
Mean TLCO<br>(range)	48<br>(18-90)	N/A
Mean FEV1<br>(range)	73<br>(40-107)	N/A
Mean CPI<br>(range)	47<br>(15-6)	N/A

Supplementary table 1

Demographic data for patient cohorts

Serum mediators cohort	IPF	Controls
n	24	11
Male	18	5
Mean age (range)	74y<br>(63-86)	69y<br>(49-86)
On antifibrotics	8 (N=1;P=7)*	N/A

Supplementary table 2

Demographic data for patient cohorts for analysis of monocyte gene expression by qPCR and for measurement of serum mediators. *N=Nintedanib, P=Pirfenidone

.

Type I IFN baseline cohort	Controls	IPF
n	10	27
Age<br>mean (range)	67<br>(56-79)	75<br>(54-84)
Male (n)	6	23
% on antifibrotics	NR	15<br>(10N, 5P*)

Supplementary table 4. Demographic for patient cohorts used for examination of type 1 IFN expression at baseline and after type I IFN stimulation. *N= Nintedanib; P=Pirfenidone
